# Circulating short chain fatty acid levels and body composition in type 2 diabetes mellitus

**DOI:** 10.7150/ijms.111920

**Published:** 2025-04-22

**Authors:** Ching-Hua Hsu, Yi-Chun Tsai, Ping-Shaou Yu, Wei-Wen Hung, Wei-Chun Hung, Shang-Jyh Hwang, Hui-Ju Tsai

**Affiliations:** 1 School of Medicine, College of Medicine, Kaohsiung Medical University, Kaohsiung, Taiwan.; 2 Department of Internal Medicine, School of Medicine, College of Medicine, Kaohsiung Medical University, Kaohsiung, Taiwan.; 3 Division of Nephrology, Department of Internal Medicine, Kaohsiung Medical University Chung-Ho Memorial Hospital, Kaohsiung Medical University, Kaohsiung, Taiwan.; 4 Department of Internal Medicine, Kaohsiung Municipal Cijin Hospital, Kaohsiung, Taiwan.; 5 Division of General Medicine, Kaohsiung Medical University, Kaohsiung Medical University Chung-Ho Memorial Hospital, Kaohsiung, Taiwan.; 6 Graduate Institute of Medicine, College of Medicine, Kaohsiung Medical University, Taiwan.; 7 Division of Endocrinology and Metabolism, Department of Internal Medicine, Kaohsiung Medical University Chung-Ho Memorial Hospital, Kaohsiung Medical University, Kaohsiung, Taiwan.; 8 Department of Microbiology and Immunology, College of Medicine, Kaohsiung Medical University, Kaohsiung, Taiwan.; 9 Department of Family Medicine, Kaohsiung Medical University Chung-Ho Memorial Hospital, Kaohsiung Medical University, Kaohsiung, Taiwan.; 10 Department of Family Medicine, School of Medicine, College of Medicine, Kaohsiung Medical University, Kaohsiung, Taiwan.; 11 Research Center for Precision Environmental Medicine, Kaohsiung Medical University, Kaohsiung, Taiwan.

## Abstract

**Background:** Short-chain fatty acids (SCFAs) may play key functional roles in the pathophysiology of type 2 diabetes (T2D) through regulating energy intake and substrate metabolism. Body composition, including fat tissue, muscle tissue and the pattern of their distribution in the body, can represent health status and be the cause or consequence of T2D complications. The aim of this study was to explore the relationship between serum SCFA levels and body composition distribution in patients with T2D.

**Methods:** This observational cross-sectional study enrolled 430 patients with T2D from October 2016 to June 2020. The levels of nine kinds of SCFAs in serum were measured using liquid chromatography mass spectrometry. Body composition, including lean tissue and fat tissue, was measured once using bioimpedance spectroscopy at enrollment.

**Results:** The mean age of the patients was 61.7 ± 12.3 years and 54.0% were male. Multivariate linear analysis revealed that the patients with the highest tertile of serum methylbutyrate level (β = -0.81, 95% CI = -1.56, -0.06, p = 0.03) and valerate/isovalerate ratio (β = -1.15, 95% CI = -1.86, -0.44, p = 0.002) had a lower fat tissue index (FTI). In subgroup analysis, the negative association of FTI with serum methylbutyrate level and valerate/isovalerate ratio was only found in the patients who were older, female, and had glycated hemoglobin ≤ 7%, urinary albumin-creatinine ratio < 30 mg/g, homeostatic model assessment for insulin resistance ≤ median value, and body mass index < 30 kg/m^2^. Conversely, none of the nine SCFAs were associated with lean tissue index.

**Conclusions:** This study found that T2D patients with a higher circulating methylbutyrate level and serum valerate/isovalerate ratio had lower FTI. The relationship was consistent in older, female patients with well-controlled glucose. Further research is needed to analyze the interactions between SCFAs and body composition with clinical metabolic outcomes in T2D patients.

## Introduction

Type 2 diabetes mellitus (T2D) is a major health issue worldwide. The prevalence of T2D is rapidly increasing globally, and is predicted to reach 629 million among people aged 20-79 years by 2045 1. T2D leads to serious complications, including retinopathy, neuropathy, cardiovascular disease, and kidney damage, significantly increasing mortality and healthcare financial burden 2. In Asia, younger populations develop T2D due to lifestyle changes and urbanization 3. There is an urgent need to identify biological mechanisms and effective approaches to manage T2D and its risk factors.

Body composition, including fat tissue, muscle tissue and the pattern of their distribution in the body, can represent health status and be a cause or consequence of T2D complications 4-6. Accumulating evidence suggests that high fat mass and low lean mass may be associated with higher levels of homeostatic model assessment for insulin resistance (HOMA-IR) 7, 8. Furthermore, the total amount of fat, its distribution, and the characteristics of adipocytes have been shown to contribute to an increase in insulin resistance, resulting in the development of T2D 9.

Short-chain fatty acids (SCFAs) are volatile fatty acids produced by intestinal bacteria to metabolize dietary fiber 10, 11. Growing evidence indicates that SCFAs have health benefits on body weight regulation, inflammatory response, and insulin sensitivity, as well as in maintaining glucose and lipid homeostasis 12-16. Available data on the effect of SCFAs in regulating body composition are mainly based on animal studies, and well-controlled studies on humans are limited. Consequently, the association between SCFAs with fat and lean tissue in T2D patients is not well-understood. Therefore, the aim of this study was to explore the relationship between serum SCFA levels and body composition in T2D patients.

## Methods

### Study participants

This observational cross-sectional study enrolled 430 T2D patients who have participated in the diabetic education program and complied with the principles of diet therapy to diabetes at Kaohsiung Medical University Hospital (KMUH) in Taiwan from October 2016 to June 2020, all of whom provided written informed consent for their participation. Patients with antibiotic use in less than one month prior to enrollment, or with inflammatory bowel disease or with surgery of the gastrointestinal tract, or with currently diagnosed cancer undergoing chemotherapy in the previous year were excluded. The study protocol was approved by the Institutional Review Board of KMUH (KMUHIRB-G(II)-20160021, KMUHIRB-G(I)-20160036, KMUHIRB-G(II)-20190036). Informed consent was obtained in written form from all of the patients, and all clinical investigations were conducted according to the principles expressed in the Declaration of Helsinki.

### Sample and clinical data collection

The patients were interviewed and their medical records were reviewed at enrollment, and information on usual dietary habits (the crude ratio of protein and fiber), history of tobacco/alcohol consumption, along with clinical data and body mass index (BMI; kg/m^2^) were recorded. The American Diabetes Association criteria were used to define diabetes in this study, as a history of the disease or the use of related medications 17. Hypertension was defined as a history of the disease or the use of related medications. Prescription data for anti-hypertensive and anti-diabetic medications and statins were also recorded. Blood and urine samples were collected after a 12-hour fast for biochemical and albuminuria analysis, respectively.

### Measurement of body composition

We used a Body Composition Monitor (BCM, Fresenius Medical Care) to measure body composition (fat and lean tissue) at enrollment. The BCM has been validated against gold-standard methods in population-based studies 18, 19. The device uses the bioimpedance spectroscopy technique at 50 frequencies ranging between 5 kHz and 1 MHz, and provides information on extracellular fluid overload and normohydrated lean/adipose tissue in the whole body 20. The fat tissue index (FTI) was used to represent normohydrated adipose tissue, and the lean tissue index (LTI) was used for normohydrated lean tissue. For the measurements, after the patients had been allowed to rest in the recumbent position for a minimum of 5 min, electrodes were placed on an ipsilateral hand and an ipsilateral foot. The analysis only included parameters that reached a quality of ≥ 95%.

### Measurement of serum SCFAs

The following SCFAs were investigated in this study: methylvalerate, butyrate, isovalerate, propionate, isobutyrate, valerate, formate, methylbutyrate, and acetate, Serum levels of these nine SCFAs were measured using liquid chromatography mass spectrometry (LC-MS). Fifty μL of serum obtained from the participants and 20 μL of 200 mM 3-nitrophenylhydrazine hydrochloride were mixed with 20 μL of 120 mM N-(3-dimethylaminopropyl)-N′-ethylcarbodiimide hydrochloride-6% pyridine in 100% aqueous methanol. After reacting for 30 min at 40℃, the solution was diluted to 210 μL with 10% aqueous methanol. Seventy-five μL of the solution was then mixed with 25 μL of the internal standard mix, and a 10 μL aliquot was used in LC-MS/MS.

All quantification and detection procedures were conducted on a Waters ACQUITY UPLC system (Waters Corporation, Milford, MA) with tandem MS (Finnigan TSQ Quantum Ultra triple-quadrupole MS, Thermo Electron, San Jose, CA) and Xcalibur software (ThermoFinnigan, Bellefonte, PA). The LC-MS/MS system was run in positive mode and used an electrospray ion source. The injection volume was 10 μL on an ACQUITY UPLC BEH C18 Column (130 Å, 1.7 µm, 2.1 mm x 100 mm, Waters) with a filter in front of the column (Waters Acquity UPLC™ BEH C18 column, 1.7 μm, 2.1 mm × 50 mm). The temperature of the column was 40°C, and the flow rate was 300 μL/min.

### Statistical analysis

The baseline characteristics of the patients were presented as continuous variables with mean ± SD or median (with 25^th^ and 75^th^ percentiles), as appropriate. Differences in continuous variables between groups were assessed using either independent t-tests or Mann-Whitney U tests, while categorical variables were compared using chi-square tests. Continuous variables with skewed distributions underwent log10 transformation to approximate normal distribution prior to further analysis. Linear or logistic regression analysis were used to assess associations between serum SCFA levels with LTI and FTI. We analyzed all clinical and laboratory factors that possibly affect body composition using the univariate analysis in Supplementary Table 1. The factors which were significant in univariate analysis (p value <0.05) were further put in multivariate analysis. We also analyzed factors that may have modified the associations between circulating SCFA levels and FTI, including sex (male/female), age (≥ 65 or < 65 years), BMI (< 30 kg/m^2^ or ≥ 30 kg/m^2^ depending on World Health Organization (WHO) criteria for obesity), median HOMA-IR value, glycated hemoglobin (HbA1c) (> 7% or ≤ 7%), and urinary microalbumin-creatinine ratio (UACR) (≥ 30 mg/g or < 30 mg/g). SPSS (version 22, IBM Inc., Armonk, NY) was used for all statistical procedures. All p-values were two-sided, with a significance threshold established at < 0.05.

## Results

### Characteristics of the patients

Table 1 shows the clinical characteristics, medication records, and laboratory parameters of the entire cohort. Of the 430 subjects, the mean age was 61.7 ± 12.3 years and 54.0% were male. The mean duration of T2D was 10.0 years, and the rates of hypertension and hyperlipidemia were 59.3% and 76.7%, respectively. The median HbA1c and UACR levels were 7.0% and 17.9 mg/g, respectively, and the mean BMI, LTI and FTI were 26.6±4.4, 11.8±2.2, and 14.5±4.5 kg/m^2^, respectively. We stratified the patients by the median values of LTI and FTI, and defined them as high and low LTI and FTI groups, respectively. The high LTI group had a significantly higher proportion of male patients, higher rates of cigarette and alcohol use, gout history, younger age, lower FTI and high-density lipoprotein (HDL) levels, and higher BMI, creatinine, and hemoglobin than the low LTI group. In addition, the high FTI group had a significantly lower proportion of male patients, lower rates of cigarette, alcohol and metformin use, lower LTI level, younger age, higher BMI, cholesterol, triglycerides, HbA1c, UACR, and prevalence of hypertension than the low FTI group.

### Serum SCFA levels and body composition in T2D patients

The median serum levels of the nine SCFAs were: formate (131.2 μM), acetate (93.7 μM), propionate (15.6 μM), butyrate (8.1 μM), isobutyrate (8.2 μM), methylbutyrate (6.6 μM), valerate (2.8 μM), isovalerate (17.4 μM), and methylvalerate (1.4 μM). The mean butyrate/isobutyrate and valerate/isovalerate values were 1.1 and 0.4, respectively. The high FTI group had a lower valerate/isovalerate ratio than the low FTI group (p = 0.004). There were no significant differences in other serum SCFA levels between the high and low LTI groups (Table 2).

We then performed linear univariate regression analysis of the determinants of LTI and FTI (Supplement Table 1). The results showed that female sex, hypertension history, BMI, total cholesterol, low density lipoprotein (LDL), log-transformed triglycerides, log-transformed UACR, serum butyrate/isobutyrate ratio (ß = 0.72, 95% confidence index (CI) = 0.01, 1.44, p = 0.04), and log-transformed isovalerate (ß = 1.19, 95% CI = 0.29, 2.09, p = 0.01) were significantly and positively associated with FTI. Age, use of cigarettes, alcohol, metformin and pioglitazone, serum albumin level, serum valerate/isovalerate ratio (ß = -2.60, 95% CI = -3.90, -1.30, p < 0.001) and log-transformed methylbutyrate level (ß = -2.16, 95% CI = -3.60, -0.72, p = 0.003) were negatively correlated with FTI. Furthermore, after adjusting age, sex and variables with a p value < 0.05 in univariable analysis for FTI including cigarettes, alcohol, hypertension history, BMI cut 30kg/m^2^, pioglitazone usage, metformin usage, serum cholesterol and albumin levels, log formed triglyceride and urine albumin/creatinine ratio, circulating log-transformed methylbutyrate level (ß = -1.41, 95% CI = -2.49, -0.34, p = 0.01) and valerate/isovalerate ratio (ß = -1.34, 95% CI = -2.32, -0.37, p = 0.007) were negatively associated with FTI (Table 3).

We further stratified the patients by tertiles of serum methylbutyrate and valerate/isovalerate. The patients with the highest tertile of methylbutyrate (β = -0.81, 95% CI = -1.56, -0.06, p = 0.03) and valerate/isovalerate (β = -1.15, 95% CI = -1.86, -0.44, p = 0.002) had a lower FTI compared to those with the lowest tertile (Table 3). However, there were no significant correlations between serum SCFA levels and LTI (Supplementary Table 1).

### Subgroup analysis for the relationship between serum SCFAs levels and FTI in T2D patients

**Figure 1:**
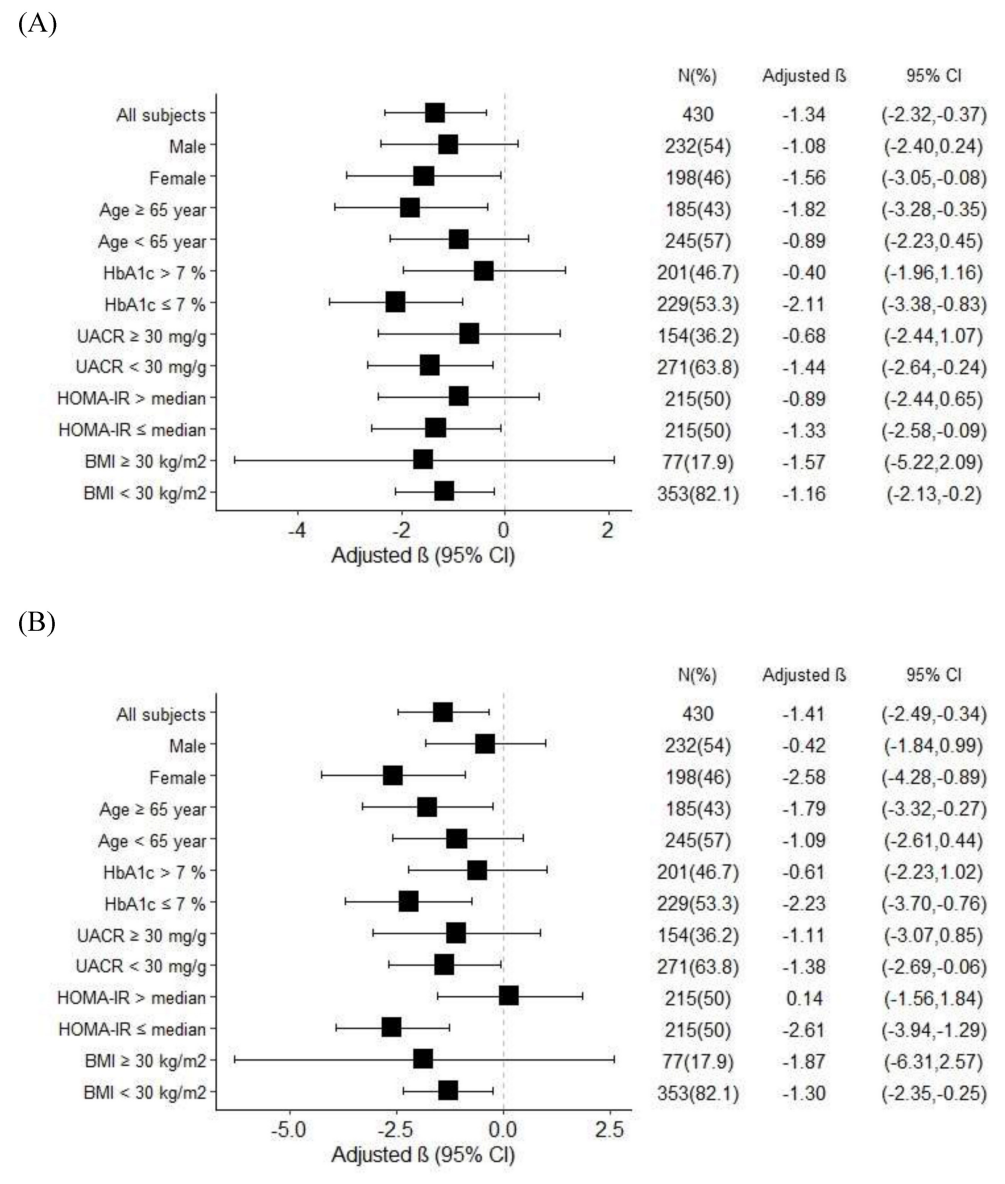
Subgroup analysis for association between. (A) Serum valerate/isovalerate ratio. (B) Serum methylbutyrate level, and fat tissue index in T2D patients.

In order to investigate the effects of age, sex, BMI, HOMA-IR, and glycemic control on FTI, we stratified the patients by age (65 years as the cut-off value for old age), sex, HbA1c (7% as cut-off value for optimal glycemic control), BMI (30 kg/m^2^ as the cut-off value for obesity), UACR (30 mg/g as the cut-off value for microalbuminuria), and HOMA-IR (median value as the cut-off value) (Figure 1). The results revealed a negative association between FTI and serum valerate/isovalerate in the patients who were female, aged ≥ 65 years, and had HbA1c ≤ 7%, UACR < 30 mg/g, HOMA-IR ≤ median value, and BMI < 30 kg/m^2^ (Figure 1A). In addition, a significant negative correlation between FTI and serum methylbutyrate was also found in the patients who were female, aged ≥ 65 years, and had HbA1c ≤ 7%, UACR < 30 mg/g, HOMA-IR ≤ median value, and BMI < 30 kg/m^2^ (Figure 1B).

## Discussion

To our best knowledge, this is the first study to explore the association between circulating SCFA levels and body composition in patients with T2D. We found that higher serum methylbutyrate level and serum valerate/isovalerate ratio were significantly correlated with lower FTI. In addition, the patients with the highest tertiles of circulating methylbutyrate and valerate/isovalerate levels had decreased fat mass after adjusting for numerous confounding factors. In subgroup analysis, the results consistently revealed a negative association between FTI with circulating methylbutyrate and valerate/isovalerate levels in the patients who were older, female, and had low insulin resistance or well-controlled glucose. Conversely, none of the nine studied SCFAs were associated with LTI.

Increasing evidence suggests that SCFAs may have a beneficial role in adipose tissue, skeletal muscle and liver substrate metabolism, thereby contributing to improved insulin sensitivity and decrease in obesity 14, 15, 21. A few studies have explored the clinical impact of branched-chain fatty acids (BCFAs) such as methylbutyrate, valerate and isovalerate 22, 23. Mika *et al.* found that twenty-three patients with excess weight presented with significantly lower serum iso-BCFA levels than twenty-one non-obese controls 24. Su *et al.* also showed that BCFA content in adipose tissue in nine persons with obesity was lower than in nine lean subjects 25. Our results showed that the patients with the highest tertiles of circulating methylbutyrate and valerate/isovalerate levels had decreased fat mass after adjusting for numerous confounding factors. In addition, we previously reported that lower circulating levels of methylbutyrate were associated with the increased severity of MASLD (metabolic-associated steatotic liver disease), and that the relationship between MASLD (metabolic-associated steatotic liver disease) severity and circulating methylbutyrate levels was independent of glucose control 23. Another study founded MASLD patients had higher plasma concentrations of valerate and α-methylbutyrate 26. Several other studies have also reported the beneficial metabolic effects of BCFAs, including improvements in insulin sensitivity and hyperlipidemia 24, 27. Pakiet *et al.* identified that insulin resistance was inversely correlated with BCFAs in 50 patients with morbid obesity 27. They also suggested that a decrease in serum BCFAs in patients with morbid obesity might be a consequence of attenuated conversion of branched-chain amino acids (BCAAs) into BCFAs in visceral adipose tissue 27. In addition, Heimann *et al.* found that isovalerate could regulate energy homeostasis through inhibiting lipolysis 28. Taken together, these findings suggest that BCFAs have the potential to be biomarkers for variations in body composition and metabolic status.

In terms of lean and fat tissue, body composition can provide a more accurate reflection of physical function and nutritional status than BMI 29. We found that serum methylbutyrate level and serum valerate/isovalerate ratio were negatively associated with FTI in the patients with well-controlled T2D. In our subgroup analysis, the patients with a BMI < 30 kg/m^2^ and higher circulating methylbutyrate level and valerate/isovalerate ratio had lower FTI, but this was not found in those with a BMI ≥ 30 kg/m^2^. We also found consistent relationships in the patients who were older, female, and with well-controlled glucose or low HOMR-IR level. In addition to age, sex, obesity, the colonic fermentation of dietary precursors of SCFAs may differ according to race, lifestyle habits, dietary habits, drugs, and co-existing diseases, thereby leading to varied abundances of circulating SCFAs 30, further affecting the relationship between SCFAs and body composition in T2D patients. For example, some oral anti-diabetic drugs such as metformin and pioglitazone, were associated with adipose tissue metabolism 31, 32. Metformin inhibits the differentiation of pre-adipocytes into mature adipocytes by downregulating adipogenic transcription factors such as peroxisome proliferator-activated receptor gamma (PPARγ) 31. Pioglitazone is a PPARγ agonist, and its primary actions target adipose tissue, which plays a central role in insulin sensitivity, lipid metabolism, and systemic inflammation 32. In our study, we have adjusted clinical characteristics including diet habit, smoke, alcohol, oral anti-diabetic drugs, hypertension, and hyperlipidemia, the correlation between SCFAs and FTI was still significant and consistent in T2D.

Adipose tissue is a key organ for the regulation of energy metabolism. It secretes a variety of peptide and protein hormones involved in the regulation of energy metabolism such as leptin and adiponectin, and pro-inflammatory cytokines such as interleukin-6 (IL-6) and tumor necrosis factor α (TNF-α) 21, 33. High fat mass has been shown to have more adverse impacts on physical function than low fat mass, and dysregulation of the production of adipokines and free fatty acids has been shown to contribute to the pathogenesis of insulin resistance, metabolic syndrome, and T2D. SCFAs induce production of the adipose tissue-derived satiety hormone leptin, and inhibit production of pro-inflammatory cytokines and chemokines 21. In addition, SCFAs may mediate the activation of free fatty acid receptor (FFAR) such as FFAR3 and FFAR2, which are expressed in intestinal, adipose, skeletal muscle, liver and pancreatic tissues 34-36, indicating that SCFAs regulate crosstalk between the gut and peripheral tissues. *In vivo* studies have reported that dietary supplementation of acetate, propionate and butyrate could prevent and treat diet-induced obesity and insulin resistance in mouse models of obesity 37, 38. These findings were explained by SCFAs increasing energy expenditure and fat oxidation via activation of AMP kinase (AMPK) and PPAR-γ coactivator (PGC)-1α in the liver and skeletal muscle 38, 39.

Previous cross-sectional studies have reported positive associations between fecal SCFA levels and obesity 40-42. A community-based study of 441 Colombian adults aged 18-62 years showed that higher fecal butyrate, acetate, propionate and total SCFA levels were positively associated with BMI, body fat, and waist circumference 40. Conversely, several other studies in Polish children and Chinese adults suggested that low fecal SCFA levels were associated with obesity 43, 44. In the current study, we found negative associations between serum SCFA levels and FTI in our cohort of patients with T2D. Because of the rapid turnover rate and dynamic variations in the blood, the concentrations of SCFAs in the blood are much lower than in feces 45. Serum SCFAs are considered to be more strongly linked with metabolic diseases than fecal SCFAs because they directly interact with the target tissues and organs through their receptors 46. In addition, few studies show that fasting serum SCFA levels are lower compared to postprandial levels 47, 48. Fasting reduces colonic fermentation activity because there is less dietary substrate available 48. This may result in lower luminal SCFA concentrations and possibly lower serum SCFA levels, although the kinetics and persistence of SCFAs in circulation are complex 48. A 12-hour overnight fast prior to blood collection is standard practice to reduce diet-induced variability in SCFA levels. Taken together, we believe that circulating SCFA levels may be an indicator of body composition in patients with T2D.

There are some limitations to this study. First, because this relatively small sample was enrolled in a single hospital, these study subjects might be insufficiently representative of the broader population. The cross-sectional study design limits the ability to establish causal relationships between SCFAs and body composition. Further study might be conduct to validate the relationship between SCFAs and body composition in another T2D cohort. Second, dietary pattern (the ratio of protein and fiber), serum SCFA, and body composition were measured at enrollment in the study. The ratio of protein and fiber intake was not significantly correlated to FTI (supplementary Table 1). However, we did not have detailed information on diet composition (such as total energy, carbohydrate, protein, and fat intake) and lifestyle, although we included the crude dietary habits of the patients. This might have led to underestimation of the impact of diet on the relationship between SCFAs and body composition. Although we adjusted these covariates including age, sex, cigarettes, alcohol, hypertension history, BMI cut 30kg/m^2^, pioglitazone usage, metformin usage, serum cholesterol and albumin levels, log formed triglyceride and urine albumin/creatinine ratio, some potential confounding factors may not be considered. Additionally, there were no nondiabetic subjects in this study to compare with the T2D patients. However, the aim of this study was to examine the relationship between circulating SCFAs and body composition in T2D patients, not in the general population. Thus, our findings were not influenced by the lack of nondiabetic subjects. In addition, we did not analyze stool levels of SCFAs to compare the differences with serum SCFAs. Further research is needed to explore the interactions among SCFAs in serum, feces and microbiota with body composition in T2D patients. Furthermore, we did not focus on consequent clinical outcomes such as cardiovascular events or mortality in this study. We will explore the consequent clinical outcome in further studies. Finally, the biological mechanism remains unknown, and future *in vitro* and *in vivo* studies are necessary to investigate the pathophysiological mechanisms of SCFAs in body composition and other novel biomarkers in T2D patients.

In conclusion, in this study of patients with T2D, those with higher circulating methylbutyrate level and serum valerate/isovalerate ratio had a lower FTI. The negative relationship between circulating methylbutyrate level and serum valerate/isovalerate ratio was only found in the patients who were older, female, and had well-controlled glucose. Further research is needed to analyze the interactions between SCFAs and body composition with clinical metabolic outcomes in T2D patients.

## Supplementary Material

Supplementary table.

## Figures and Tables

**Table 1:** The characteristics of study participates stratified by the median of lean tissue index and fat tissue index

	Entire cohort (n=430)	LTI < median (n=216)	LTI > median (n=214)	P-value	FTI < median (n=215)	FTI > median (n=215)	P-value
Age, year	61.7±12.3	63.5±10.9	60.1±11.5	0.002	63.5±10.5	60.1±11.9	0.002
Sex (male), %	54.0	20.3	87.9	<0.001	68.8	39.1	<0.001
Smoke, %	22.8	10.6	35.0	<0.001	29.3	16.3	0.001
Alcohol, %	19.5	8.3	30.8	<0.001	26.5	12.6	<0.001
Hypertension, %	59.3	61.6	57.0	0.34	54.9	63.7	0.04
Gout, %	7.0	2.3	11.7	<0.001	7.0	7.0	0.58
Hyperlipidemia, %	76.7	77.3	76.2	0.78	80.0	73.5	0.07
DM duration, year	10.0±8.3	10.6±9.0	9.1±7.6	0.11	10.3±8.3	9.4±8.3	0.26
Body Mass Index, kg/m^2^	26.6±4.4	25.8±4.1	27.3±4.6	<0.001	23.8±2.5	29.4±4.2	<0.001
Diet habit, %							
Protein more than fiber	16.3	10.3	22.5	<0.001	15.2	17.4	0.642
Fiber more than protein	33.1	42.1	24.9		31.9	34.2	
Fiber equal to protein	50.6	47.6	53.6		52.9	48.4	
**Medication**							
Sulfonylurea (yes v.s. no)	42.8	45.8	39.7	0.20	41.4	44.2	0.31
DPP4 inhibitor (yes v.s. no)	64.7	63.9	65.4	0.74	65.6	63.7	0.38
Metformin (yes v.s. no)	84.4	81.9	86.9	0.16	87.9	80.9	0.03
SGLT2 inhibitor (yes v.s. no)	2.8	1.5	4.2	0.11	3.1	2.6	0.51
Pioglitazone (yes v.s. no)	30.0	28.2	31.8	0.42	32.6	27.4	0.15
Insulin (yes v.s. no)	15.1	16.7	13.6	0.37	14.0	16.3	0.30
Statin (yes v.s. no)	44.9	46.3	43.5	0.55	43.7	46.0	0.35
**Body composition**							
Lean mass index, kg/m^2^	11.8±2.2	10.0±1.2	13.6±1.3	<0.001	12.4±2.0	11.2±2.2	<0.001
Fat mass index, kg/m^2^	14.5±4.5	15.6±4.4	13.5±4.5	<0.001	11.1±1.8	18.0±3.8	<0.001
**Laboratory parameters**							
Cr, mg/dl	1.0±0.5	0.8±0.4	1.1±0.4	<0.001	1.0±0.3	0.9±0.4	0.14
Albumin, mg/dl	4.5±0.3	4.5±0.3	4.6±0.4	0.07	4.6±0.3	4.5±0.3	0.06
Hemoglobin, g/dl	13.7±1.7	13.2±1.5	14.3±1.6	<0.001	13.8±1.7	13.6±1.6	0.22
Cholesterol, mg/dl	169.6±39.9	171.1±42.0	165.4±37.6	0.14	164.4±24.839.0	172.1±40.5	0.04
Triglyceride, mg/dl	122(86,179)	118.5(91.0,159.8)	118.5(80,188)	0.06	103(72,160)	136(96,196)	<0.001
HDL, mg/dl	45.5±17.6	48.3±15.4	42.3±16.8	<0.001	46.2±17.6	44.5±15.1	0.28
LDL, mg/dl	95.7±32.8	97.7±35.2	93.7±29.7	0.21	91.9±30.6	99.5±34.1	0.02
HbA1C, %	7.0(6.5,8.0)	7.8±4.7	7.3±1.3	0.15	6.9(6.3,7.5)	7.2(6.6,8.3)	<0.001
Urinary ACR, mg/g	17.9(6.6,68.0)	20.4(7.8,69.8)	13.3(5.1,63.3)	0.42	12.3(5.1,49.0)	21.9(8.1,87.6)	0.001

Abbreviations: DPP4, Dipeptidyl peptidase 4; ACEI, angiotensin converting enzyme inhibitors; ARB, angiotensin II receptor blockers; Cr, creatinine; HbA1C, glycated hemoglobin; HDL, high-density lipoprotein; LDL, low desity-lipoprotein; ACR, albumin-creatinine ratio; LTI, lean mass index; FTI: fat mass index; SGLT2, sodium-glucose cotransporter 2; DM: diabetes mellitus

**Table 2:** Serum SCFA distribution of study participates stratified by the median value of lean mass index (LTI) and fat mass index (FTI)

	Entire cohort (n=430)	LTI < median (n=216)	LTI > median (n=214)	P-value	FTI < median (n=215)	FTI > median (n=215)	P-value
Formate	131.2(93.1,217.9)	131.3(94.6,218.3)	129.7(92.2,217.9)	0.28	132.1(97.5,216.6)	131.2(89.1,221.7)	0.28
Acetate	93.7(73.5,131.7)	93.9(74.4,125.3)	93.0(71.1,135.8)	0.84	97.2(73.1,139.3)	89.1(73.8,121.8)	0.08
Propionate	15.6(11.6,21.5)	15.7(11.7,21.7)	15.4(11.4,21.5)	0.84	16.5(11.5,21.8)	15.3(11.6,21.1)	0.48
Butyrate	8.1(5.5,9.8)	8.1(5.8,9.8)	1.2(0.4,1.5)	0.76	8.1(5.1,9.8)	8.1(6.0,9.7)	0.39
Isobutyrate	8.2(5.6,12.9)	8.2(5.5,12.5)	8.3(5.6,13.4)	0.42	8.7(5.8,13.9)	7.2(5.5,12.2)	0.12
Butyrate/Isobutyrate	1.1±0.6	1.1±0.6	1.0±0.6	0.29	1.0±0.6	1.1±0.6	0.11
Methylbutyrate	6.6(4.7,14.1)	6.5(4.7,14.3)	6.5(4.6,13.8)	0.88	7.7(4.7,15.2)	6.2(4.5,12.6)	0.13
Valerate	2.8(1.7,5.3)	2.7(1.7,5.3)	2.8(1.7,5.3)	0.68	2.9(1.7,5.3)	2.6(1.6,5.0)	0.74
Isovalerate	17.4(3.6,24.9)	17.9(3.9,26.3)	16.9(3.1,24.3)	0.25	14.8(3.2,23.9)	18.6(4.1,26.3)	0.05
Valerate/Isovalerate	0.4±0.3	0.4±0.3	0.4±0.3	0.53	0.5±0.3	0.4±0.3	0.004
Methylvalerate	1.4(0.7,3.3)	1.4(0.6,3.3)	1.5(0.8,3.3)	0.70	1.5(0.7,3.3)	1.4(0.7,3.3)	0.88

**Table 3:** Linear regression of serum SCFAs and fat tissue index

	Crude B(95%Cl)	P-value	Adjusted B(95%CI)	P-value
Butyrate/isobutyrate	0.72(0.01,1.44)	0.04	0.22(-0.30,0.74)	0.41
Butyrate/isobutyrate tertile 1	reference		reference	
Butyrate/isobutyrate tertile 2	0.93(-0.13,1.98)	0.09	-0.37(-1.11,0.36)	0.32
Butyrate/isobutyrate tertile 3	0.88(-0.06,2.05)	0.06	0.13(-0.59,0.84)	0.73
Log (Methylbutyrate)	-2.16(-3.60,-0.72)	0.003	-1.41(-2.49,-0.34)	0.01
Methybutyrate tertile 1	reference		reference	
Methybutyrate tertile 2	-0.45(-1.50,0.59)	0.39	-0.07(-0.79,0.64)	0.84
Methybutyrate tertile 3	-1.66(-2.71,-0.62)	0.002	-0.81(-1.56,-0.06)	0.03
Log (Isovalerate)	1.19(0.29,2.09)	0.01	0.33(-0.30,0.96)	0.30
Isovalerate tertile 1	reference		reference	
Isovalerate tertile 2	0.43(-0.63,1.48)	0.42	0.10(-0.61,0.81)	0.78
Isovalerate tertile 3	1.37(0.32,2.42)	0.01	0.36(-0.38,1.09)	0.33
Valerate/Isovalerate	-2.60(-3.90,-1.30)	<0.001	-1.34(-2.32,-0.37)	0.007
Valerate/Isovalerate tertile 1	reference		reference	
Valerate/Isovalerate tertile 2	-0.22(-1.27,0.84)	0.69	-1.00(-1.83,-0.18)	0.02
Valerate/Isovalerate tertile 3	0.95(-0.11,2.00)	0.07	-1.15(-1.86,-0.44)	0.002

Adjusted model: age, sex, smoke, alcohol, hypertension history, BMI cut 30kg/m^2^, pioglitazone usage, metformin usage, serum cholesterol and albumin levels, log formed triglyceride and urine albumin/creatinine ratio.
