# Supplementary material for: Circulating short chain fatty acid levels and body composition in type 2 diabetes mellitus
Source: Int J Med Sci. 2025 Apr 22;22(10):2289–97. doi: 10.7150/ijms.111920 (PMC12080568; doi:10.7150/ijms.111920)
Supplement: Supplementary file 1 — Supplementary table. [file ijmsv22p2289s1.pdf]

Supplement Table 1. Linear and logistic univariate regression of determinants of lean tissue index and fat tissue index

	Lean tissue index		Lean tissue index >median		Fat tissue index		Fat tissue index >median	
	Crude ß (95%Cl)	P-value	Crude OR(95%Cl)	P-value	Crude ß (95%Cl)	P-value	Crude OR(95%Cl)	P-value
Clinical data								
Age, year	-0.05(-0.07,-0.03)	<0.001	0.97(0.96-0.99)	0.002	-0.09(-0.13,-0.05)	<0.001	0.97(0.95-0.99)	0.002
Sex (female *v.s.* male)	-2.99(-3.30,-2.68)	<0.001	0.04(0.02-0.06)	<0.001	2.63(1.80,3.45)	<0.001	3.44(2.31-5.13)	<0.001
Body mass index, kg/m^2	0.11(0.07,0.16)	<0.001	1.09(1.04-1.13)	<0.001	0.90(0.86,0.95)	<0.001	1.94(1.71-2.19)	<0.001
Smoke (yes *v.s.* no)	1.31(0.83,1.79)	<0.001	4.53(2.70-7.58)	<0.001	-1.47(-2.48,-0.45)	0.005	0.47(0.29-0.75)	0.001
Alcohol (yes *v.s.* no)	1.62(1.12,2.12)	<0.001	4.91(2.79-8.61)	<0.001	-1.61(-2.69,-0.54)	0.003	0.40(0.24-0.66)	<0.001
Hypertension (yes *v.s.* no)	-0.19(-0.62,0.24)	0.38	0.83(0.56-1.22)	0.34	1.26(0.39,2.13)	0.005	1.44(0.98-2.13)	0.06
Statin usage (yes *v.s.* no)	-0.18(-0.60,0.24)	0.40	0.89(0.61-1.30)	0.55	0.19(-0.68,1.06)	0.66	1.10(0.75-1.61)	0.63
Metformin (yes *v.s.* no)	0.48(-0.09,1.06)	0.10	1.46(0.86-2.48)	0.16	-1.45(-2.63,-0.27)	0.02	0.58(0.34-1.00)	0.05
Pioglitazone (yes *v.s.* no)	0.11(-0.35,0.56)	0.63	1.18(0.78-1.79)	0.42	-1.25(-2.19,-0.32)	0.009	0.78(0.52-1.19)	0.25
Diet habit								
Fiber equal to protein	-	-	-	-	-	-	-	-
Protein more than fiber	0.51(-0.08,1.09)	0.09	1.95(1.10-3.45)	0.02	1.09(-0.14,2.32)	0.08	1.25(0.72-2.15)	0.43
Fiber more than protein	-0.82(-1.28,-0.36)	<0.001	0.51(0.33-0.78)	0.002	0.20(-0.77,1.17)	0.69	1.17(0.77-1.80)	0.46
Laboratory data								
Creatinine, mg/dl	1.21(0.71,1.72)	<0.001	4.64(2.51-8.54)	<0.001	-0.34(-1.41,0.73)	0.53	0.70(0.43-1.13)	0.15
Albumin, g/dl	1.10(0.47,1.73)	0.001	1.73(0.95-3.16)	0.07	-1.81(-3.12,-0.50)	0.007	0.56(0.31-1.03)	0.06
Hemoglobin, g/dl	0.49(0.37,0.61)	<0.001	1.65(1.44-1.90)	<0.001	0.03(-0.29,0.23)	0.81	0.93(0.83-1.04)	0.22
Cholesterol, mg/dl	-0.00(-0.01,0.00)	0.21	0.99(0.99-1.00)	0.14	0.02(0.00,0.03)	0.007	1.01(1.00-1.01)	0.04
Log (Triglyceride)	0.52(-0.34,1.38)	0.23	1.37(0.63-2.99)	0.43	4.74(3.02,6.46)	<0.001	8.82(3.72-20.88)	<0.001
HDL, mg/dl	-0.02(-0.04,-0.01)	<0.001	0.96(0.95-0.98)	<0.001	0.00(-0.03,0.03)	0.90	0.99(0.98-1.01)	0.30
LDL, mg/dl	-0.00(-0.01,0.00)	0.29	0.99(0.99-1.00)	0.21	0.02(0.01-0.03)	0.002	1.01(1.00-1.01)	0.02
Log UACR, mg/g	-0.23(-0.49,0.04)	0.10	0.87(0.68-1.11)	0.27	1.05(0.50,1.59)	<0.001	1.46(1.14-1.88)	0.003
HbA1C, %	-0.03(-0.10,0.03)	0.26	0.91(0.80-1.04)	0.17	0.06(-0.07,0.19)	0.35	1.19(1.04-1.37)	0.01
SCFA								
Log (Formate)	-0.20(-0.80,0.41)	0.53	0.91(0.53-1.57)	0.73	-0.22(-1.47,1.04)	0.73	0.99(0.58-1.73)	0.99
Log (Acetate)	0.48(-0.63,1.59)	0.40	1.09(0.40-2.96)	0.87	-1.99(-4.28,0.30)	0.09	0.35(0.12-0.98)	0.04
Log (Propionate)	0.04(-0.92,0.99)	0.94	0.71(0.30-1.70)	0.44	-0.67(-2.64,1.31)	0.51	0.80(0.34-1.90)	0.61
Log (Butyrate)	0.17(-0.86,1.19)	0.75	0.69(0.27-1.74)	0.43	0.87(-1.25,2.98)	0.42	1.45(0.57-3.67)	0.43

Log (Isobutyrate)	0.33(-0.58,1.24)	0.47	1.36(0.59-3.12)	0.46	-1.80(-3.68,0.09)	0.06	0.59(0.26-1.36)	0.22
Butyrate/Isobutyrate	-0.07(-0.42,0.28)	0.70	0.85(0.62-1.16)	0.30	0.72(0.01,1.44)	0.04	1.30(0.94-1.78)	0.11
Log (Methylbutyrate)	0.13(-0.58,0.83)	0.73	0.99(0.52-1.87)	0.97	-2.16(-3.60,-0.72)	0.003	0.57(0.30-1.08)	0.08
Log (Valerate)	0.08(-0.48,0.65)	0.77	0.87(0.52-1.45)	0.60	-0.37(-1.54,0.81)	0.54	0.93(0.56-1.55)	0.78
Log (Isovalerate)	0.01(-0.44,0.45)	0.97	0.81(0.54-1.20)	0.29	1.19(0.29,2.09)	0.01	1.55(1.04-2.32)	0.03
Valerate/Isovalerate	0.13(-0.51,0.77)	0.69	1.21(0.67-2.15)	0.53	-2.60(-3.90,-1.30)	<0.001	0.43(0.24-0.77)	0.005
Log (Methylvalerate)	0.32(-0.17,0.81)	0.20	1.05(0.67-1.64)	0.83	-0.58(-1.60,0.45)	0.27	0.95(0.61-1.48)	0.80
